# Experience of Orthodontic Treatment and Symptoms of Temporomandibular Joint in South Korean Adults

**Published:** 2018-01

**Authors:** Sang-Hee HWANG, Shin-Goo PARK

**Affiliations:** 1. Dept. of Dentistry, School of Medicine, Keimyung University, Daegu, South Korea; 2. Dept. of Occupational and Environmental Medicine, School of Medicine, Inha University, Incheon, South Korea

**Keywords:** Orthodontic treatment, Temporomandibular joint

## Abstract

**Background:**

No epidemiological studies have targeted the association between experience of orthodontic treatment and symptoms of temporomandibular joint (TMJ) in a large adult population. In this study, we investigated whether experience of orthodontic treatment is associated with symptoms of TMJ in adults.

**Methods:**

We used data from the Fifth Korea National Health and Nutrition Examination Survey (KNHANES V), conducted in 2011. Trained dentists asked subjects to report their experience of orthodontic treatment and symptoms of TMJ. Overall, 5936 subjects aged over 19 yr were included in this study (2528 males). The data were analyzed using the chi-square test and multiple logistic regression tests.

**Results:**

The group with experience of orthodontic treatment had more symptoms of TMJ than the group without orthodontic experience. After adjusting for all covariates (i.e., age, sex, marital status, income, education, stress, teeth injury, and occupation), the adjusted odds ratio was 2.53 (95%CI 1.74–3.67).

**Conclusion:**

Experience of orthodontic treatment could be related to increased symptoms of TMJ.

## Introduction

Temporomandibular disorders (TMDs) are a group of conditions that involve the chewing muscles, the temporomandibular joints (TMJ), and related tissues, whose symptoms are diverse (1).

Symptoms such as pain, limited opening, asymmetrical movement of the jaw, and joint sounds are the most common findings (2).

Associated factors with TMDs have been reported to be psychological (anxiety, stress), structural (occlusion), functional (bruxism), genetic factors, hyperlaxity, orthodontic treatment, and external traumas (3, 4).

Many previous studies, including systemic reviews, have been done on the association between orthodontic treatment and TMDs. Most of them have found no relationship between orthodontic treatment and TMDs. However, those previous studies examined mainly children or adolescents. No epidemiological studies of the subject have targeted a large population of adults (5–7).

Recently, the number of adults wanting orthodontic treatment has been increased because of the change in the recognition of aesthetic orthodontic treatment, the development of new aesthetic orthodontic techniques (i.e., hidden braces or clear orthodontic aligners), and the increased orthodontic possibility helping prosthetic rehabilitation. The change in patient age has increased the possibility of patients presenting with symptoms of TMJ (6).

We investigated the association between experience of orthodontic treatment and symptoms of TMJ in Korean adults using a representative sample of Koreans from the Fifth Korea Health and Nutrition Examination Survey in 2011 (KNHANES V).

## Methods

We used data from KNHANES V, conducted in 2011. KNHANES is a nationwide survey drawn from different census populations and housing units that consider the proportion of each subgroup. The survey is carried out by the Centers for Disease Control (CDC) of Korea to obtain statistically reliable and representative data on the health, food, and lifestyles of the Korean population.

The Institutional Review Board approved KNHANES V for Human Subjects of the Korea CDC (2011-02CON-06-C).

Out of 10589 candidates, 8055 (76.1%) participated in the study. Oral inspections were administered to adults over 19 yr of age (n=6566). Excluding 630 subjects who provided improper answers, we analyzed the results of 5936 subjects (2528 males).

The experience of orthodontic treatment was based on the question, “Have you ever received orthodontic treatment so far?” with responses of “Yes” or “No.” Trained dentists posed this question to all subjects to identify whether or not the absence of first or second premolar is due to caries before the clinical examination in the oral health survey.

All the questions of the symptoms of TMJ were prepared by specialists based on the criteria by WHO (8, 9). Each subject was interviewed to assess the symptoms of TMJ. Trained dentists asked the subjects the following three questions. 1. Have you ever noted any clicking sound present with mouth opening over the past year? 2. Have you ever experienced pain or tenderness in the temple, in the ear, or in front of ear over the past year? 3. Have you ever suffered pain or limitation with mouth opening, or jaw unlocked over the past year? Trained dentists defined the symptoms of TMJ when the subjects answered yes to at least one of the questions regarding the symptoms of TMJ.

We performed Chi-square test to compare differences in Symptoms of TMJ associated with socio-demographic characteristics, and we calculated odds ratios (OR) using a multiple logistic regression models adjusted for potential confounders.

To identify the correlation between experience of orthodontic treatment and symptoms of TMJ, we investigated the factors of age, sex, marital status, income, education, stress, teeth injury, implants, and occupation as potential confounders. Trained interviewers asked participants for them. For example, the stress was based on the question “How much stress do you have in daily life?” with responses of “much” or “less”. Trained dentists examined the teeth injury and implants. We used SPSS for Windows (version 12.0; SPSS Inc., Chicago, IL, USA) for all analyses and set the level of statistical significance at 0.05.

## Results

People with increased symptoms of TMJ tended to be young, female, single, well educated, highly stressed and have a tooth injury or white-collar occupation (Table 1).

**Table 1:** General characteristics and Symptoms of temporomandibular joint

***General characteristics***	***Symptoms of TMJ***			***OR^a^***	***95%CI***

	**No, n (%)**	**Yes, n (%)**	***P*-value**		
Age (yr)					
≤29	546(82.5)	116(17.5)	<0.001	1	1
30 – 39	956(89.2)	116(10.8)		0.664	0.457–0.966
40 – 49	971(94.1)	61(5.9)		0.403	0.260–0.625
50 – 59	1099(95.7)	49(4.3)		0.296	0.182–0.482
≥60	1968(97.3)	54(2.7)		0.180	0.104–0.312
Sex					
Male	2392(94.6)	136(5.4)	0.001	1	
Female	3148(92.4)	260(7.6)		1.470	1.159–1.865
Marital status					
Married	4870(94.8)	268(5.2)	<0.001	1	
Single	663(83.9)	127(16.1)		1.456	1.020–2.077
Income					
Low	1268(93.3)	91(6.7)	0.048	1	
Lower middle	1381(92.6)	111(7.4)		1.076	0.796–1.456
Upper middle	1447(94.8)	80(5.2)		0.725	0.524–1.004
Upper	1388(92.5)	112(7.5)		1.009	0.739–1.377
Education					
≤elementary	1461(96.7)	50(3.3)	<0.001	1	
Middle school	609(96.1)	25(3.9)		0.860	0.502–1.473
High school	1790(92.6)	143(7.4)		0.932	0.589–1.473
≥college	1552(90.1)	171(9.9)		1.157	0.709–1.886
Stress					
Less	4020(94.2)	249(5.8)	<0.001	1	
Much	1394(90.8)	141(9.2)		1.372	1.095–1.718
Teeth injury					
No	5451(93.4)	385(6.6)	0.023	1	
Yes	66(86.8)	10(13.2)		1.660	0.799–3.448
Implant					
No	5067(93.2)	367(6.8)	0.290	1	
Yes	463(94.5)	27(5.5)		1.019	0.667–1.558
Occupation					
White	1707(91.3)	162(8.7)	<0.001	1	
Blue	1464(95.4)	71(4.6)		1.046	0.750–1.458
Unemployed	2240(93.5)	156(6.5)		1.036	0.801–1.340

a Outcomes adjusted for variables in each model

Table 2 shows the odds ratio (95% confidence interval, CI) from the multiple logistic regression analyses of the association between experience of orthodontic treatment and symptoms of TMJ. The group with experience of orthodontic treatment had more symptoms of TMJ than the group without orthodontic experience. After adjusting for all covariates, the adjusted OR was 2.53 (95%CI 1.74–3.67).

**Table 2:** Odds ratios and confidence intervals for Symptoms of TMJ according to experience of orthodontic treatment

***Experience of Orthodontic treatment***	***Symptoms of TMJ***		***Crude odds ratio***		***Adjusted odds ratio***	
	**No, n (%)**	**Yes, n (%)**	**OR**	**95%CI**	**OR^a^**	**95%CI**
No	5390 (94.0)	345 (6.0)	1		1	
Yes	138(73.4)	50 (26.6)	5.66	4.02–7.96	2.53	1.74–3.67

a Adjusted by age, sex, marital status, income, education, stress, teeth injury, and occupation

## Discussion

This study is the first to evaluate the correlation between experience of orthodontic treatment and symptoms of TMJ among adults aged ≥19 yr using a large population.

It confirmed across all adults aged ≥19 yr that experience orthodontic treatment could be related to increased symptoms of TMJ.

Our finding remained significant after adjusting for socio-demographic variables.

This does not necessarily mean that an experience of orthodontic treatment is associated with clinical TMDs. Despite the relative commonness of TMJ symptoms, only approximately 10% of cases require treatment (3). The TMJ sounds rarely progress to severe clinical problems and some researchers have suggested that they may be a normal condition, rather than an illness; hence unnecessary treatment of TMJ sounds should be avoided (10, 11). A clinical TMDs diagnosis requires a proper examination of the masticatory muscles, articular disks, soft tissues of the TMJ, type of pain, and mandibular functional movements, and analysis of patients’ behavior should be considered (12).

There has been much confusion over the correlation between orthodontic treatment and temporomandibular disorders (TMDs) among adults. Reasons for this confusion may include: the population that wants braces and the population that get TMDs are similar (the majority of both being females aged 20s and 30s), making this cause-effect relationship an easy conclusion; the simplistic understanding of the rigor required to establish cause and effect; poor research conclusions drawn from small, poorly devised (often uncontrolled) studies or studies based on opinion and anecdotes (13).

Although the relationship remains controversial, we hypothesize reasons to explain a potential relationship between experience of orthodontic treatment and symptoms of TMJ in adults. Patients with malocclusion are more likely than those without to receive orthodontic treatment. Alterations in occlusion could be the predisposing, triggering, or perpetuating factors of TMDs (3). However, a relatively weak association has been observed between occlusal factors and TMDs, and most published studies have used a cross-sectional design. Few firm conclusions can be drawn regarding a possible causal relationship (3).

The hypothesis was tested orthodontic treatments that achieve good occlusal parameters have different relationships with TMDs in adults from those that do not. Within the subset of individuals who underwent previous orthodontic treatment, there were no clinically relevant differences in the presence of TMDs between the subjects with a history of ideal orthodontics and those with non-ideal orthodontics. The only exception was the potential increased risk for TMJ disc displacement described in individuals received non-ideal orthodontic treatment (14). Edgewise straight wire orthodontic treatment involving Class II elastics have no effect, or little effect (i.e. mild pain “lateral to TMJ capsule”), on TMJ sign and symptoms (15).

A meta-analysis study revealed consistent results among 38 primary studies. No study indicated that traditional orthodontic treatment increased the prevalence of TMDs except for mild signs (soft click) (16). One study reporting mild signs compared a group of previously treated orthodontic patients with a control group, and eight variables were selected to assess the joint dysfunction and occlusal stability. No significant association was observed between the groups and the signs or symptoms except in the case of soft clicks of the joints, where there was a higher percentage in the treatment group (16). We speculate that click sound could be main reason for increased symptoms of TMJ in our study.

Because orthodontic treatment lasts approximately two years, orthodontic patient may have symptoms of TMJ for other reasons during that time. However, unsatisfied patients may misunderstand and believe their symptoms of TMJ were caused by orthodontic treatment.

The present study had the following limitation. Trained dentists inquired about the symptoms of TMJ over the past year, which could result in an overestimation. On the other hand, this is unlikely because the results in the present study (6.7%) were similar to those of another study (10.8%) in subjects aged 35–44 yr (17). Because it is a cross-sectional study, causal relationships cannot be identified. An ongoing follow-up study is needed to determine the causal relationships between experience of orthodontic treatment and symptoms of TMJ in adults.

## Conclusion

Experience of orthodontic treatment could be related to increased symptoms of TMJ.

## Ethical considerations

Ethical issues (Including plagiarism, informed consent, misconduct, data fabrication and/or falsification, double publication and/or submission, redundancy, etc.) have been completely observed by the authors.
